# Metabolomics-based combination of GH and NVB in the treatment of NSCLC lung cancer recurrence

**DOI:** 10.7150/jca.102722

**Published:** 2025-01-01

**Authors:** Xinxin Li, Fengfei Chen, Rui Yang, Zhaohui Song, Xiaohui Ma, Li Sun, Shengtao Yuan

**Affiliations:** 1 Jiangsu Key Laboratory of Drug Screening, China Pharmaceutical University, Nanjing, 210009, China.; 2 National Key Laboratory of Chinese Medicine Modernization, Tasly Academy, Tasly Holding Group Co. Ltd., Tianjin, 300410, China.

**Keywords:** Ginsenoside H dripping pills, Vinorelbine, Postoperative Recurrence, Metabonomics, Alkaline Phosphatase

## Abstract

Lung cancer is one of the most harmful cancers in the world, endangering the lives and health of many people. Although there are various methods to treat lung cancer at present, but lung cancer is asymptomatic in the early stages and has a high recurrence rate after late treatment which make it difficult to cure with conventional treatments. Drug combinations for the treatment of lung cancer have been used in many clinical studies. In this study, we constructed a recurrence model of Non-Small Cell Lung Cancer (NSCLC) lung cancer and used a combination of Ginsenoside H dripping pills (GH) and vinorelbine (NVB) to treat the recurrence of lung cancer. The results showed that the inhibition rate of the combined treatment of GH and NVB is 74.81% which is significantly higher than the therapeutic effect of separate use. We also used GC-TOF/MS-based metabolomics to identify differentially abundant metabolites in relapse models and explore biomarker trends. We found that there are 12 metabolite differences in the abundance of metabolites between the treatment groups (GH group, NVB group and GH-NVB group) and the model group, such as glucose 6-phosphate, palmitoleic acid, linoleic acid, guanine, allantoic acid. The differences in these metabolites involve glucose and lipid metabolism, amino acid metabolism, and purine metabolism. We further analyzed the changes in the content of these metabolites and found that the combined use of GH and NVB can regulate purine metabolism, folate synthesis, and thiamine metabolism, ultimately reducing the abnormal increase in alkaline phosphatase (AP). This study provides a method for the treatment of lung cancer and some biomarkers for the detection of lung cancer.

## Introduction

Lung cancer is a malignancy that originates in the bronchial mucosa or glands of the lungs 1 and is the first most common cancer and the leading cause of cancer death 2. Many factors can contribute to the development of lung cancer, such as occupational exposure 3, ionizing radiation 4, diet 5, genetics 6, and lung disease 7; 8. There are many methods to treat lung cancer, such as surgery 9, radiotherapy and chemotherapy 10, immunotherapy 11 and molecular targeted therapy 12. And surgical resection has always been the main method of treating lung cancer 13. However, the high recurrence rate, strong drug resistance and easy diffusion of lung cancer have greatly reduced the survival rate of lung cancer patients 14. More importantly, patients with early-stage lung cancer usually have no obvious symptoms 15 which results in many patients being diagnosed only when the cancer has developed to an advanced stage. This delay greatly reduces the effectiveness of lung cancer treatment and the success rate of cancer treatment.

In order to solve the drug resistance of tumors in cancer treatment, enhance the efficacy of drugs and reduce the toxicity of single drug doses, combined drugs have been developed as a new technology for treating cancer 16. Drug combinations have great potential in cancer treatment and have been successfully used in various cancers, such as breast cancer 17, esophageal cancer 18, gastric cancer 19 and lung cancer 20. Some combination drugs combined with chemotherapy to treat lung cancer have been used in the treatment of patients with advanced lung cancer, such as TP (Paclitaxel (PTX) + Cisplatin (DDP)) 21 and NP (Vinorelbine (NVB) + DDP) 22. However, various side effects may occur when these combination drugs are combined with chemotherapy to treat lung cancer patients, such as loss of appetite, fatigue, vomiting and nausea 23. Therefore, it is necessary to develop new drug combinations to prevent postoperative recurrence or metastasis and reduce side effect.

Ginsenoside H Dropping Pills (GH) is a drug used for the adjuvant treatment of advanced Non-Small Cell Lung Cancer (NSCLC) 24, and the main component of the drug is ginsenoside Rh2. Ginsenoside Rh2 has anti-tumor activity and immunomodulatory effects and is widely used in the treatment of various tumors, such as liver cancer 25 and lung cancer 26. Moreover, ginsenoside Rh2 is also used as an auxiliary drug for chemotherapy and radiotherapy, and some studies have also combined ginsenoside with other drugs for tumor research 27. A study on the combination of GH and cyclophosphamide (CTX) can improve the quality of life of lung cancer patients after surgery by reversing the Th1/Th2 shift caused by CTX 24.

The difficulty of early detection of lung cancer and the high recurrence rate after treatment of late-stage lung cancer bring many difficulties to the treatment of lung cancer. In this study, we combined GH and NVB to treat lung cancer recurrence, and we also used GC-TOF/MS (Gas Chromatography-Time-of-Flight Mass Spectrometry)-based metabolomics to identify differentially abundant metabolites in the recurrence model and explore biomarker trends during the treatment of lung cancer. This study provides a combined drug solution for the treatment of lung cancer recurrence. It also explores the changing trends of biomarkers during the treatment of lung cancer, which can provide important clinical reference. These biomarkers can not only serve as predictors of lung cancer recurrence, but can also be used to evaluate treatment effects and monitor disease progression in patients.

## Methods

### Materials and animals

The experimental animals in this study were 10-week-old adult male C57BL/6 mice purchased from Vital River Laboratory Animal Technology Co., Ltd. (Beijing, China). The lung cancer cells used in this study were Lewis Lung Carcinoma (LLC) purchased from ATCC (American Type Culture Collection). And Lewis lung cancer cells are a model of NSCLC. Animal care and surgical procedures were all guided by the Animal Care and Control Committee of China Pharmaceutical University.

The two pharmaceutical reagents used in this study are Ginsenoside H dripping pills and NVB, they are from the Modern TCM research platform (Tasly Group, Tianjin, China) and Haosen Pharmaceutical Co., Ltd. (Jiangsu, China) respectively. And ginsenoside H dripping pills contain 71% ginsenoside Rh2.

### Animal postoperative recurrence model

The experimental animals in this study were 10-week-old C57BL/6 mice. We injected 1 × 10^6^ Lewis cells subcutaneously into the right front axilla of 48 mice to construct a mouse model of lung cancer, and selected 8 mice to be injected with an equal volume of PBS solution as a control group. For mice inoculated with Lewis cells, we will regularly use calipers to measure the size of the mouse tumors, and determine the tumor volume through the equation LW^2^/2 (L represents the length of the tumor, W represents the width of the tumor). When the tumor volume of mice reached 500 mm^3^, we randomly divided the cancer-inoculated mice into 4 groups (8 mice in each group), namely model group, GH group, NVB group and GH-NVB group. The control group of this study was normal mice injected with PBS solution, while the model group was mice injected with Lewis cells and then subjected to surgical resection without drug treatment.

We performed resection surgery on these four groups of mice with axillary tumors according to the resection surgery protocol of Predina *et al.* 28 to construct an animal postoperative recurrence model. We anesthetized the mice in the model group, GH group, NVB group and GH-NVB group by intraperitoneal injection of chloral hydrate (400 mg/kg), and shaved the hair at the tumor site with a hair clipper before surgery. A 1-1.5 cm incision was made near the tumor, and 90% of the tumor tissue was removed by surgical shear. Finally, mycelia-free 4-0 suture was used to close the incision. To avoid wound laceration in mice, we fed each mouse individually.

### Dosage regimen

To avoid the mice's wounds opening up during administration, we started dosing these mice two days after surgery. The mice in the control group, model group and NVB group were given 0.5% CMC-Na (Carboxymethyl Cellulose Sodium) (0.1ml/10g) every day (Table S1), while the GH group and GH-NVB group were given GH dissolved in 0.5% CMC-Na (36 mg/kg, 0.1ml/10g). For the NVB group and GH-NVB group, we also gave NVB (4.15 mg/kg, 0.1ml/10g) dissolved in normal saline every week. For the control group, model group and GH group, we gave the same amount of normal saline (0.1ml/10g) to each group.

### Evaluation of postoperative recurrence

Fourteen days after administration to five groups of experimental mice, we weighed the mice and killed them by cervical dislocation. We surgically removed tumors from the model group, GH group, NVB group and GH-NVB group and weighed these tumors. We evaluated recurrence inhibition (E) by comparing the tumor weight of the three drug administration groups with the tumor weight of the model group. We used the following formula to evaluate relapse inhibition in the drug administration group: E = (W_M_-W_A_) / W_M_ ×100% (W_M_ represents the tumor weight of the model group, and W_A_ represents the drug administration group). The significance between the administration group and the model is *p* < 0.05 and the inhibition rate is greater than 30%, indicating that the drug has valuable anti-cancer activity.

In order to further understand the effect of the combination of NVB and GH in the treatment of lung cancer, we used the cooperation index (q) to evaluate the therapeutic effect of the combination of NVB and GH. The calculation formula of q is: q=E_AB_ / [E_A_ + (1-E_A_) E_B_] (E_A_, E_B_, and E_AB_ are the recurrence inhibition rates of the GH, NVB, and GH-NVB groups respectively). According to the evaluation method of the combination of drugs 29, q <0.85 indicates antagonism between the two drugs, q>1.15 indicates synergy, and 0.85< q <1.15 indicates additive effect.

### Evaluation of quality of life

In this study, we evaluated the quality of life of mice in different groups by calculating the net body weight and immune organ index (IOI) 30 of five groups of mice. The net body weight (NBW) of the mouse was calculated by subtracting the weight of the cancer tissue from the body weight of the mouse. For mouse immune organ index, we dissected the spleen and thymus from mice, washed and weighed them. We used Worgan/NBW (Worgan represents the weight of mouse spleen and thymus) to calculate the IOI of mice.

### Untargeted metabonomics analysis

We collected serum from 5 groups of mice for metabolomic analysis. Blood samples were collected intravenously from mice treated 14 days after administration, incubated in a 37℃ water bath, centrifuged at 2500 rpm for 15min to separate serum samples, and immediately stored in liquid nitrogen for subsequent serum biochemical index detection. These serum samples were pretreated according to the methods 31; 32, and the metabolites were extracted for metabolomic analysis. In this study, we used a GC‒TOF‒MS (Gas Chromatography-Time-of-Flight Mass Spectrometry) system (Pegasus HT, Leco Corp., St. Joseph, MO, USA) equipped with an Rxi-5MS column (30 m×250 μm, 0.25μm film thickness; Restek corporation, Bellefonte, PA, USA) for metabolite analysis. GC-MS is more suitable for analyzing volatile and semi-volatile small molecules. Some metabolites in serum have better separation effect and detection sensitivity in GC-MS analysis.

We used XploreMET v3.0 (Metabo-Profile, Shanghai, China) to process and analyze the data obtained from chromatography-mass spectrometry. We used principal component analysis (PCA) 33 to reduce the dimensionality of the data to compare the intrinsic relationships between extracted metabolites in different groups. We also used partial least squares discriminant analysis (PLS-DA) 34 and orthogonal partial least squares discriminant analysis (OPLS-DA) 35 to filter out irrelevant information from specified groups to maximize the identified metabolic differences.

We used XploreMET software to determine the structure and characteristics of metabolites by comparing retention index and spectral data with data in the JiaLib metabolite database. For the identified differential metabolites, we conducted functional analysis and biological significance analysis. We further analyzed the metabolic pathways of these metabolites. We used KEGG (http://www.genome.jp/kegg/) 36 and HMDB (http:/ /www.hmdb.ca/) 37 database for metabolic pathway analysis. Finally, MetaboAnalyst 3.0 38 was used to integrate relevant metabolic pathways and obtain pathways significantly related to lung cancer.

### ELISA detection of serum alkaline phosphatase

To validate the hypothesis derived from the analysis of potential biomarkers, alkaline phosphatase (AP) levels in serum and tumor tissue of each group were measured using an ELISA kit. An alkaline phosphatase detection kit was purchased from Nanjing Jiancheng Bioengineering Institute (Jiangsu, China). We homogenized tumor tissue in phosphate-buffered saline (PBS) to detect protein concentration and then AP levels.

### Statistical analysis

All results are presented as the mean ± SD. GraphPad Prism 8 was used to analyze the data. One-way ANOVA was performed to evaluate the statistical significance of differences between multiple groups. A value of *p <0.05* was considered to indicate a significant difference.

## Results

### The therapeutic effect of GH and NVB combined with recurrence of lung cancer

**Figure 1:**
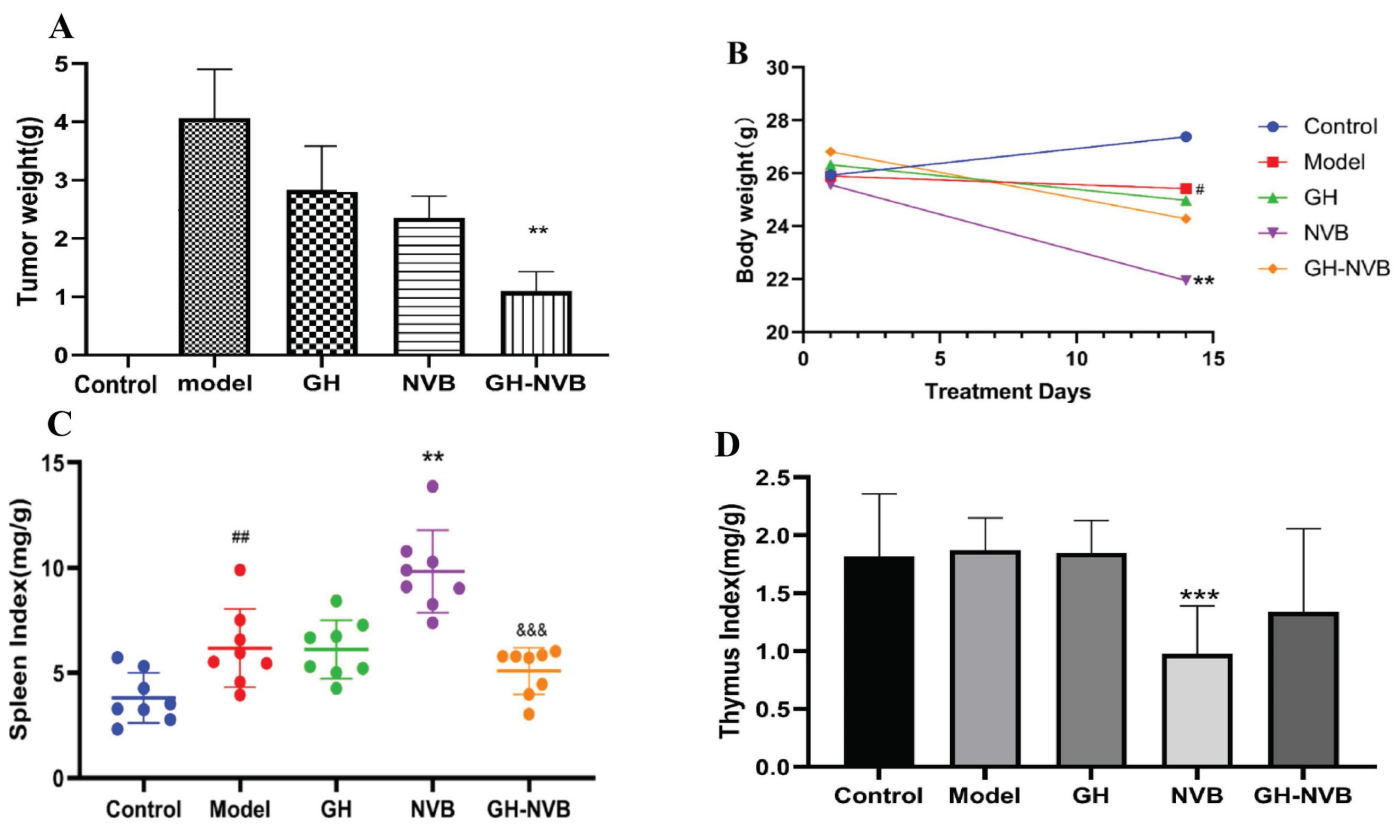
The combination of GH and NVB had synergistic effects on tumor recurrence and quality of life *in vivo*. A: Statistical analysis of tumor weight and the related images. B, C, D: Body weight, spleen index and thymus index in different groups indicated a prominent improvement in quality of life. ## *p* <0.01 vs. Control; ** *p* <0.01 vs. Model; *** *p* <0.001 vs. Model; &&& *p* <0.001 vs. NVB.

In this study, we first explored the therapeutic effect of GH and NVB alone on surgical resection of lung cancer. We counted the tumor weights of the control group, model group, GH group, NVB group and GH-NVB group respectively, which were 0, 4.05g, 2.85g, 2.53g and 1.02g respectively. The result has found that using GH and NVB alone has a certain effect on inhibiting recurrence after surgical resection, and the inhibition rates for lung cancer are 30.63% and 37.53% respectively (Figure 1A). The combined treatment of GH-NVB group in the treatment of recurrence after surgical resection of lung cancer (inhibition rate 74.81%) was better than that used alone, and had a significant inhibitory effect compared with the model (P < 0.01, Figure 1A). And the tumor weights of GH-NVB group were significantly lower than the tumor weights of GH group and NVB group (P < 0.05). We further calculated the synergy index q of GH and NVB (q=1.16, q>1.15), indicating that the combination of GH and NVB has a synergistic anti-relapse effect. This result shows that the use of GH combined with NVB has a good general effect in the treatment of lung cancer.

To further understand the effects of GH and NVB treatment on the quality of life of mice after surgical resection, we calculated the net body weight (Figure 1B), spleen index (Figure 1C), and thymus index (Figure 1D) of the mice in the five groups. The average weight of the control group, model group, GH group, NVB group and GH-NVB group are 27.5, 25.8, 25.6, 22.1 and 25.2 g. The spleen index of the control group, model group, GH group, NVB group and GH-NVB group are 4.2, 6.1, 6.0, 8.2 and 5.5 mg/g. The thymus index of the control group, model group, GH group, NVB group and GH-NVB group are 1.8, 1.9, 1.9, 0.9 and1.3 mg/g.

Compared with the control group, the model group showed changes in net weight loss (*p* < 0.05) and splenomegaly (*p* < 0.01). Compared with the model group, the NVB group had a significant decrease in net body weight (*p* < 0.01) and thymus atrophy (*p* < 0.001), but splenomegaly (*p* < 0.01) which might be caused by the toxic side effects of NVB itself. There were no significant changes in net body weight, spleen index, and thymus index between the GH group and the model group. This result also showed that the therapeutic effect of GH alone on cancer is relatively weak, and GH is usually used as an auxiliary drug in clinical practice. The net body weight, spleen index or thymus index of the GH-NVB group all showed a downward trend compared with the model group, but there was no significant correlation. Compared with the NVB group, the spleen index in the GH-NVB group decreased significantly (P<0.001), and net body weight, weight gain, and thymus index also showed an improvement trend that indicating that NVB toxicity was improved after combined treatment.

### Metabolite differential analysis

**Figure 2:**
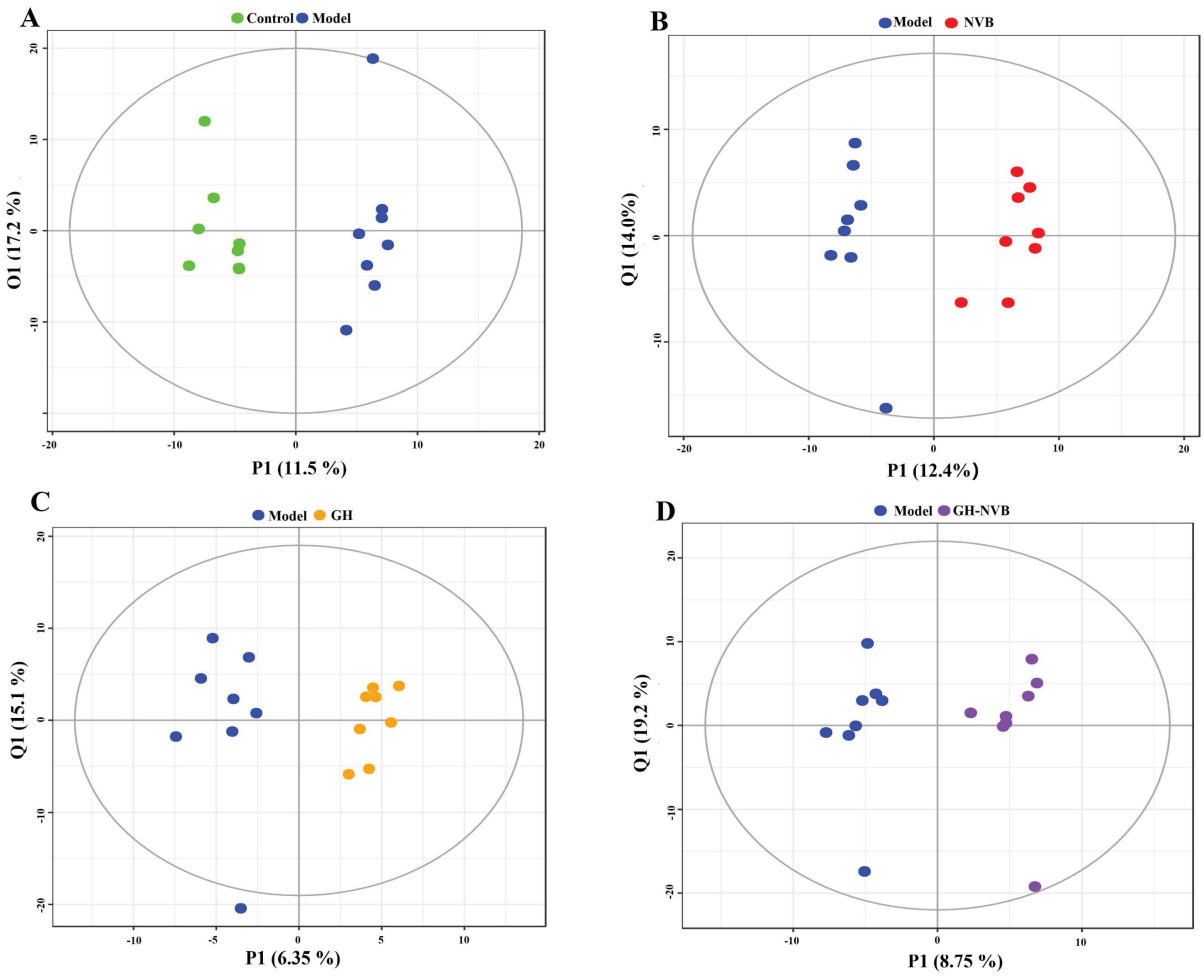
Metabolic profiles of the individuals from groups were further differentiated using a statistical Model. A: OPLS-DA Model of the control vs. Model group. B: OPLS-DA Model of the NVB vs. the Model group. D: OPLS-DA Model of the GH group vs. the Model group. H: OPLS-DA Model of the GH-NVB group vs. the Model group.

**Figure 3:**
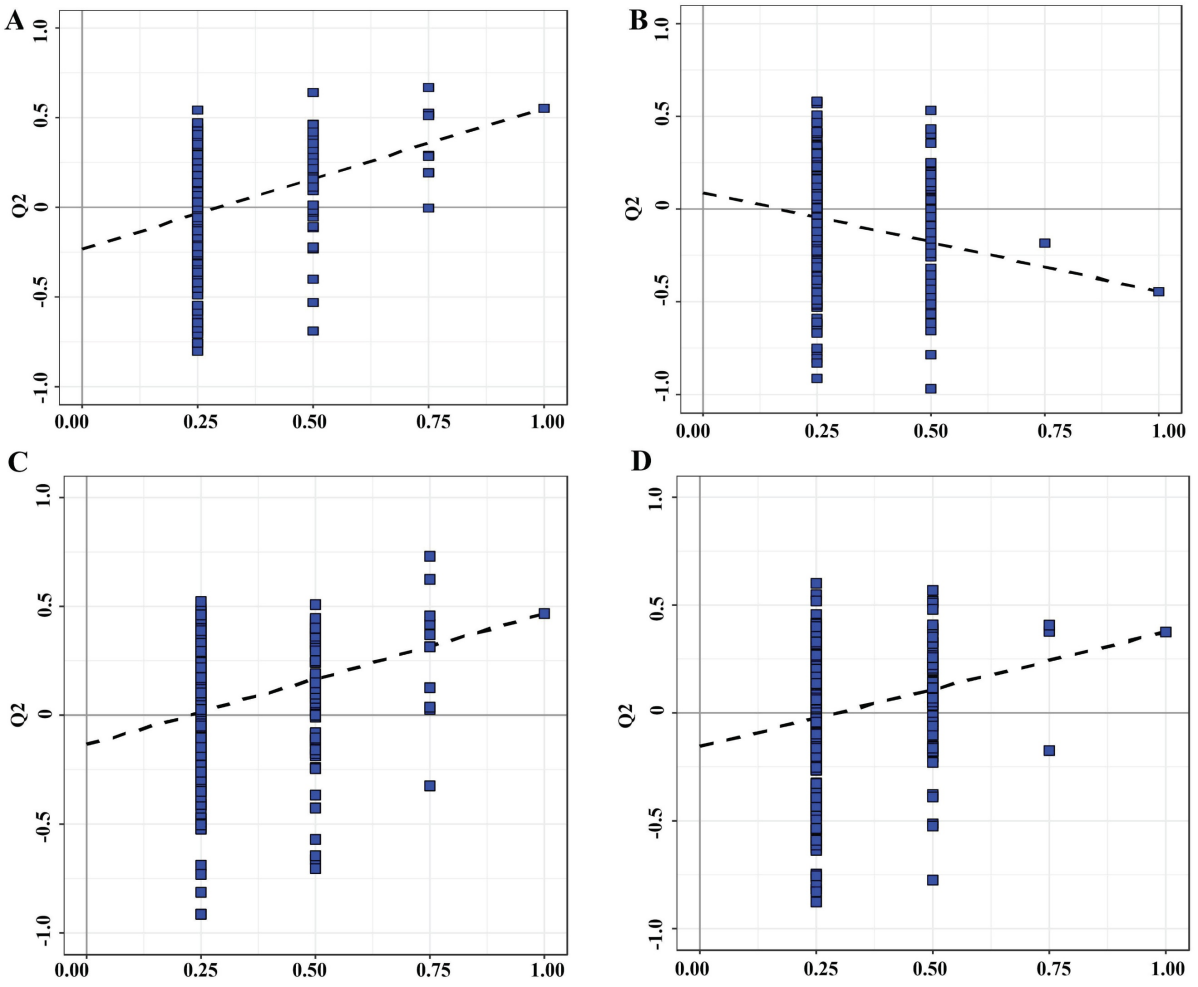
The substitution test evaluates the validity of the classification model. A: OPLS-DA Model of the control vs. Model group. B: OPLS-DA Model of the NVB vs. the Model group. D: OPLS-DA Model of the GH group vs. the Model group. H: OPLS-DA Model of the GH-NVB group vs. the Model group.

In order to further understand the differences in metabolic profiles between the relapse model group and the control group treated with GH and NVB combined, we performed PCA analysis of metabolites in the serum of five groups of mice (Figure 2, Figure 3). Except for a blue point in the GH and control groups that is located in the standard deviation area (Figure 2C), the points in the other groups are all within the area. These indicate that the model in the study is reliable and that the samples involved in the model are correct for subsequent analysis. Moreover, serum samples in the control group and model group, model group and GH group, model group and NVB group, and model group and GH-NVB group all showed clearly separated aggregation communities, and the differences were significant.

We used the intersection of multivariate statistics and univariate statistics to identify differences in metabolite abundance between the treatment group and the model group, and we identified 12 metabolites with differential abundance between control and administration groups (GH group, NVB group and GH-NVB group) (Table 1). It was found that there were 12 metabolites of different abundance between GH group and model group, among which inositol was down compared with model group. Compared with the model group, the NVB group had 6 differentially rich metabolites, among which citric acid, gamma-aminobutyric acid, linoleic acid and palmitic acid were down-regulated. There were 5 differentially rich metabolites between GH-NVB group and model group. These metabolites play an important role in biological processes such as cell signaling, cell cycle regulation, and apoptosis, which in turn play a key role in the development of cancer. This result suggests that these metabolites might be involved in the development of lung cancer. Certainly, the role of these substances in the development of lung cancer needs further experimental verification.

### Metabolic pathway enrichment analysis

**Figure 4:**
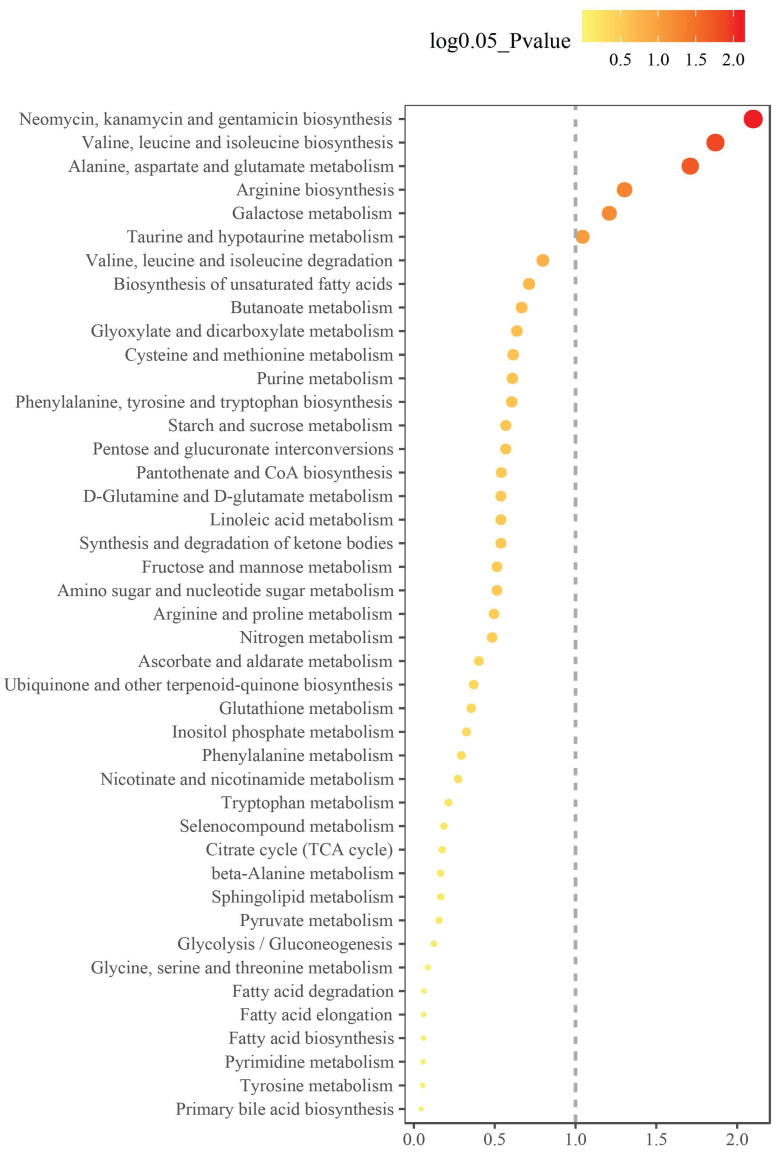
Metabolic pathways of metabolites that were significantly altered between the GH-NVB group and the model group.

To understand the metabolic pathways involved in the differentially abundant metabolites in the GH-NVB group and the model group, we performed enrichment analysis on the metabolic pathways involved in these metabolites (Figure 4). Enrichment analysis results show that these metabolites are mainly involved in the synthesis of some substances, such as alanine, aspartate and glutamate metabolism (Figure S2), valine, leucine and isoleucine biosynthesis (Figure S3), arginine biosynthesis (Figure S4).

In Neomycin, kanamycin and gentamicin biosynthesis, these substances play key roles in cancer therapy, such as inducing tumor cell apoptosis, anti-tumor immune activity and inhibiting tumor angiogenesis 39; 40. However, in this study, we found that drug-treated lung cancer can affect the metabolic pathways of neomycin, kanamycin and gentamicin *in vivo*, thereby increasing the therapeutic effect. In Valine, leucine and isoleucine biosynthesis, the synthesis of Ketoleucine increased in the administration group, while the synthesis of L-leucine and L-valine decreased. This result indicates that these metabolic pathways play an important role in the occurrence and development of lung cancer. Moreover, the synthesis of some substances may be related to lung cancer treatment, which may become a potential target for lung cancer treatment.

### Trending biomarker hypothesis analysis

**Figure 5:**
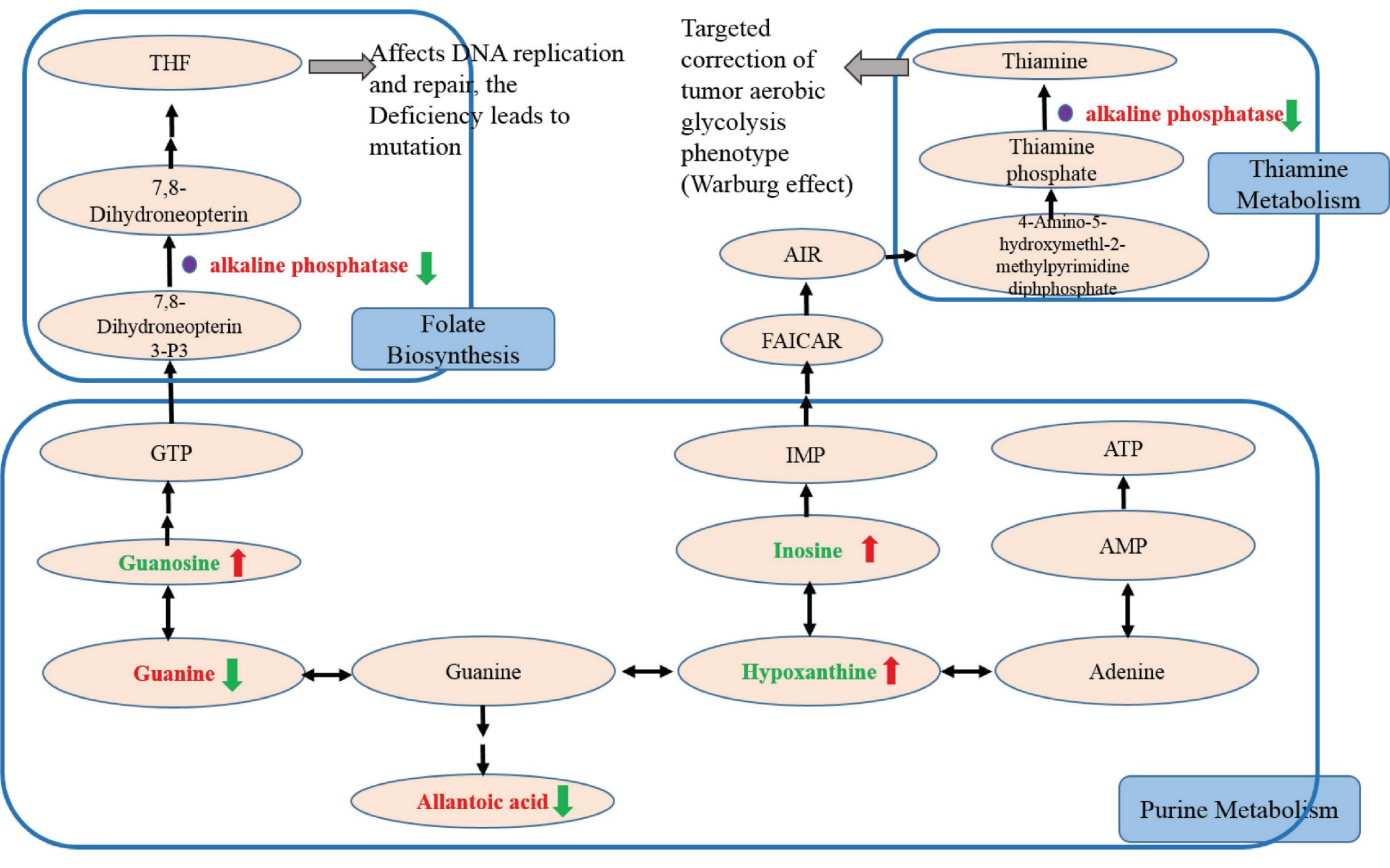
Analysis of trending biomarkers. The red text indicates that compared with the control group, the metabolites of the model group, GH group, NVB group, and GH-NVB group were up-regulated. Green indicates that compared with the control group, the metabolites of the model group, GH group, NVB group, and GH-NVB group were down-regulated. The red arrow indicates that compared with the model group, the metabolites in the GH-NVB combination group were up-regulated. The green arrow indicates that compared with the model group, the metabolites of the GH-NVB combination group are down-regulated.

In this study, we further studied potential biomarkers related to purine metabolism to discover the trend of biomarkers related to NSCLC recurrence and the related mechanism of action of GH combined with NVB (Figure 5). Compared with the control group, it was found that the Guanine, alkaline phosphatase and allantoic acid contents of the model group, GH group, NVB group, and GH-NVB group increased, while the Guanosine, Inosine and Hypoxanthine contents decreased.

Compared with the model group, it was found that in the GH-NVB group guanine and guanine/guanosine ratios were significantly increased, guanosine was significantly decreased, and allantoic acid was also significantly increased. Based on the analysis of related metabolic pathways (purine pathway) and the results of the above metabolites, we speculate that guanine is not converted into guanosine, but is accumulated in large amounts and converted into xanthine, and xanthine is metabolized into allantoic acid, resulting in allantoic acid Raise levels. Moreover, our results also found that hypoxanthine and hypoxanthine/inosine ratios were also significantly reduced in the administration group. This result suggests that the conversion of xanthine to hypoxanthine is also reduced, driving further increases in allantoic acid levels. The reduction of the metabolite GTP produced by the conversion of guanine to guanosine will lead to the reduction of raw materials for the conversion of Guanosine Triphosphate (GTP) into Tetrahydrofolate (THF), thus affecting the downstream folate biosynthetic pathway. Studies have shown that the accumulation of AP occurs due to reduced THF synthesis, leading to the upregulation of AP.

In addition, we compared the guanine/guanosine ratio between the model group and the GH-NVB group. Our guanine/guanosine ratio in the GH-NVB group was significantly lower than that in the model group (P=0.083, FC=0.577), while hypoxanthine and inosine levels were significantly increased. These results indicate that the conversion of guanine to GTP or hypoxanthine is increased and allantoic acid levels are also significantly decreased during the treatment of lung cancer recurrence by GH and NVB. There was no significant difference in serum phosphate concentration between the model group and the control group, indicating that AP participates in related reactions resulting in an increase in phosphate levels (such as reaction products). In the GH-NVB group, GH-NVB regulated folate biosynthesis and thiamine metabolism to a normal state, thereby contributing. AP can serve as a trend biomarker related to purine metabolism, folate biosynthesis, and thiamine metabolism.

**Figure 6:**
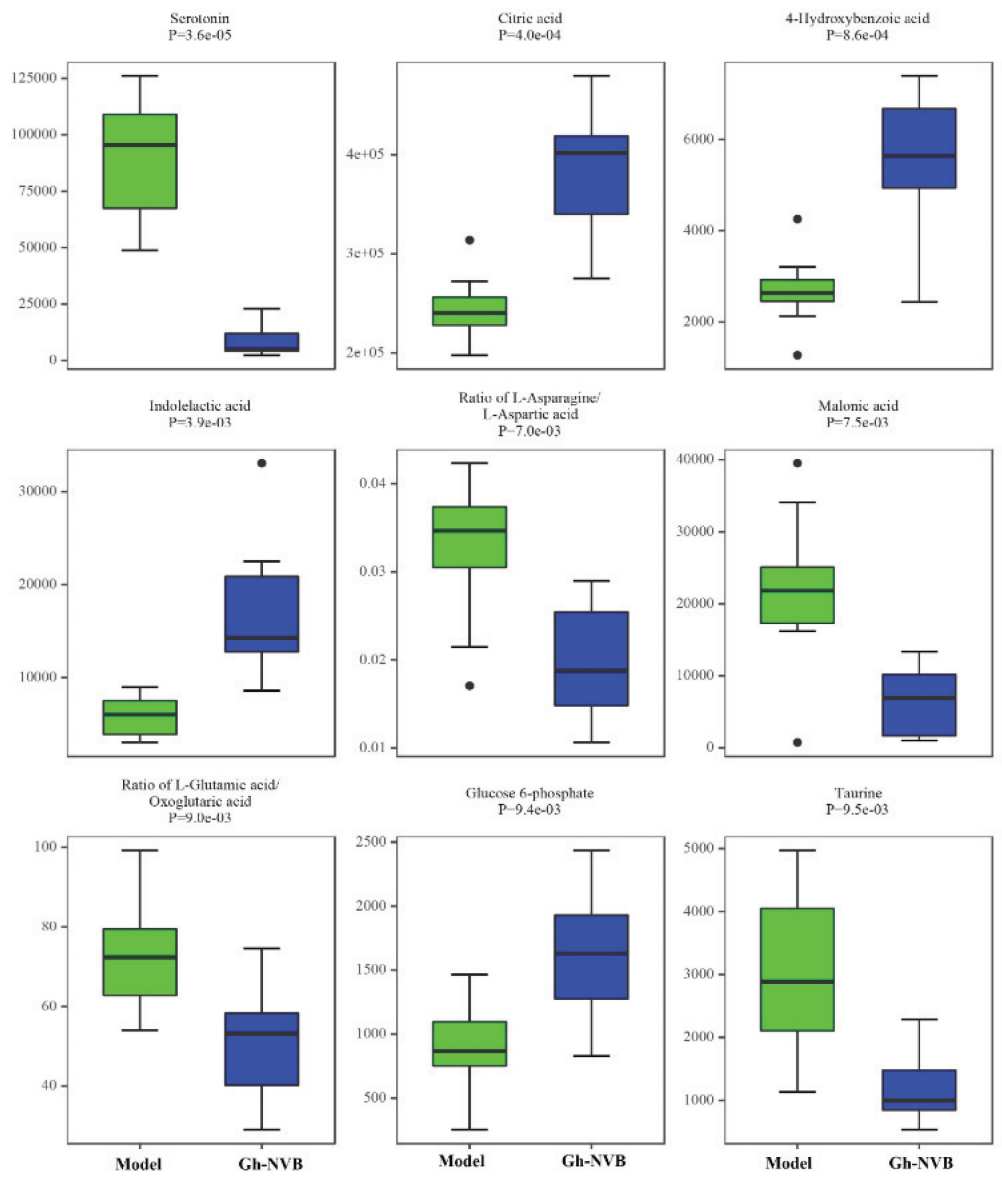
The content of nine metabolites in the GH-NVB group and the model group. Green represents the model group and blue represents the GH-NVB group.

The treatment of pulmonary recurrence model by GH and NVB is related to purine metabolism, folate biosynthesis and thiamine metabolism. We further analyzed the differences in the contents of 9 metabolites related to these three metabolic pathways in the model group and the administration group (Figure 6). We found that there were obvious differences in the contents of these 9 metabolites between the model group and the drug treatment group, such as serotonin, citric acid, 4-hydroxybenzoic acid, indolelactic acid and glucose 6-phosphate. This result further verifies the association between GH combined with NVB in the treatment of lung cancer recurrence and purine metabolism.

### Regulation of alkaline phosphatase in serum and carcinoma tissues

**Figure 7:**
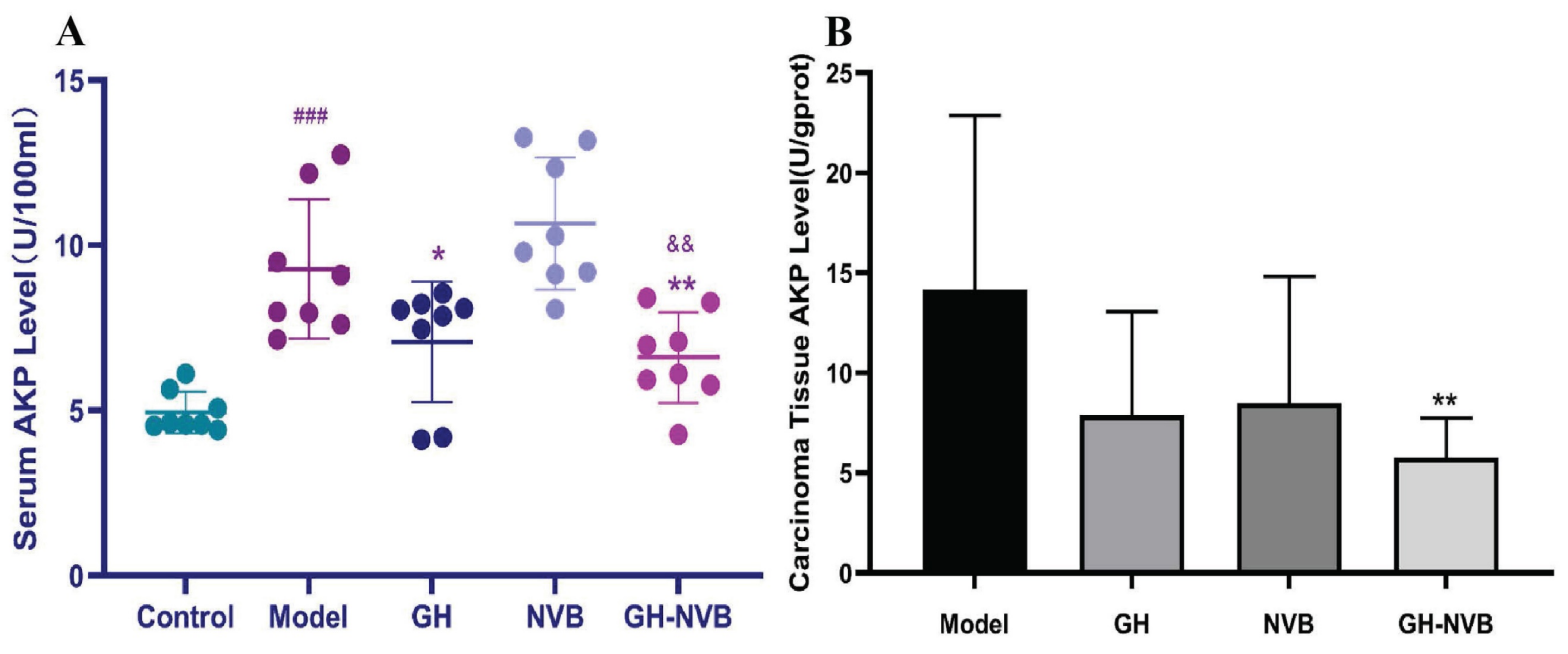
Detection of AP levels in serum and carcinoma tissues. ### *p* <0.001 vs. Control; * *p* <0.05 vs. Model; ** *p* <0.05 vs. Model.

Through the results of the metabolome, we have found that the AP content in the administration group was significantly higher than that in the model group. We further used a kit to detect the AP content in the serum (Figure 7). The results showed that the AP levels in the model group and drug administration group were significantly higher than those in the normal control group (*p* < 0.001). Compared with the model group, AP activity in the GH group and GH-NVB group was significantly reduced (*p* < 0.05 and *p* < 0.01). The AP level of the NVB group was similar to that of the Model group. This means that NVB is mainly related to the metabolism of palmitic acid and linoleic acid, but not to purine metabolism, which is related to AP. This result indicates that NVB is not involved in regulating AP levels in the purine metabolism pathway, and GH may increase the therapeutic effect of NVB by participating in the purine metabolism pathway.

## Discussion

### Postoperative treatment of lung cancer

Lung cancer is one of the malignant tumors that poses the greatest threat to human health and life safety 27, and it is also a common malignant tumor with the fastest growing morbidity and mortality in the world. Clinically, surgical treatment is often recommended for lung cancer 41. The 5-year postoperative survival rate can reach more than 70%, but more than 20% of patients still die due to postoperative recurrence and metastasis. Recurrence and metastasis after lung cancer surgery have become an important factor affecting the prognosis of patients 42.

Studies have shown that GH has anti-tumor activity, and GH can inhibit the proliferation and invasion of tumor cells and induce tumor cell apoptosis 43. Moreover, GH has been found to regulate the immune system and enhance the body's immune function 44. GH can promote the activation and proliferation of immune cells, enhance cell-mediated immune responses, and thereby enhance the body's resistance to tumors 45; 46. Some studies have explored the combined use of GH with chemotherapy drugs, these studies show that GH can enhance the efficacy of chemotherapy drugs, reduce the toxic side effects of chemotherapy, and improve the survival rate of patients 47. Especially in the treatment of NSCLC, the combined application of GH and chemotherapy drugs (such as gemcitabine, cisplatin) has shown clinical effects 48.

Postoperative adjuvant therapy is crucial for reducing recurrence, prolonging survival, and improving quality of life after complete resection of early- and mid-stage NSCLC tumors^49^. Currently, adjuvant chemotherapy, particularly cisplatin-based double-drug regimens that include NVB 50, gemcitabine 51, PTX 52, nab-paclitaxel 53, pemetrexed 54 and etoposide 55 is a primary treatment for lung cancer. However, these drugs often have serious side effects, and the survival benefit from adjuvant chemotherapy is modest, with only about a 5% increase in the 5-year survival rate. Recent advancements in medical technology have led to the development of new treatment strategies to enhance efficacy and reduce side effects 56. While NVB effectively inhibits tubulin assembly to block mitosis and prevent cancer cell proliferation, it can also cause bone marrow suppression, gastrointestinal issues, peripheral nerve toxicity, and venous toxicity 57. Combining GH with PD-1/PD-L1 inhibitors has shown efficacy in clinical trials which can improve survival rates in advanced NSCLC patients 58. Additionally, GH in conjunction with targeted therapies (gefitinib) has demonstrated promise in cancer research 59. GH not only enhances anti-cancer effects but also mitigates the toxicity of chemotherapy drugs like NVB. Its strong antioxidant capacity helps remove excess free radicals, reducing oxidative stress and protecting normal cells from chemotherapy-induced damage. Furthermore, GH's anti-inflammatory properties inhibit the release of inflammatory factors that can lessen inflammation-related toxicity 24.

Combinations of cancer drugs can significantly enhance treatment effectiveness 60. Combination chemotherapy employs drugs with different mechanisms of action, reducing tumor cell resistance and maximizing tumor growth inhibition 61. By targeting multiple survival and proliferation pathways simultaneously, these combinations improve therapeutic outcomes and lower the likelihood of drug-resistant cancer cells 62. Natural substances have been widely studied and applied as combination drugs for cancer. Many natural substances have diverse anticancer activities and can inhibit tumor growth and spread through different mechanisms, they are considered as ideal candidates for combination therapy 63. Additionally, compounds with immunomodulatory properties, like polysaccharides and peptides, can boost the immune system's ability to identify and eliminate tumors that can improve cancer treatment 64. Most of the ingredients in GH drugs are natural substances, including ginsenoside Rh2. In the field of cancer treatment, ginsenoside Rh2 has been found to inhibit the proliferation and metastasis of tumor cells and induce tumor cell apoptosis 65. Studies have shown that ginsenoside Rh2 can exert its anti-tumor effects through multiple mechanisms, including inhibiting the proliferation of tumor cells, inducing apoptosis, inhibiting angiogenesis, and regulating the tumor microenvironment 43; 66. The results of this study also indicate that GH can enhance the cancer therapeutic effect of NVB.

### Metabolome after lung cancer surgery

The study of cancer metabolome 67 reveals the metabolic processes in the occurrence, development and treatment of cancer by analyzing the metabolic differences between cancer cells and normal cells. This programming of metabolic processes often involves changes in energy metabolism, biosynthetic pathways, and signaling pathways 68. These changes contribute to cancer cell proliferation, invasion, and metastasis. Carbon metabolism is critical to cellular metabolism 69 and is closely related to folate, purine, and adenosine. Recent studies have shown that DNA methylation is an important hub of carbon metabolism and plays a key role in cellular life. As an important intermediate, THF plays an important role in maintaining normal chromosome conformation and DNA methylation 70. THF and folate cycle intermediates, along with purines and adenosine, regulate the growth and proliferation of cancer cells. Thiamine (vitamin B1) is an important cofactor in energy metabolism pathways such as sugar, fatty acid and amino acid metabolism 71. And it is essential for maintaining normal metabolism, cell growth and proliferation. It can also promote gastrointestinal motility and increase appetite. Thiamine can inhibit the growth of tumor cells in an anaerobic environment 72, so supplementing thiamine during radiotherapy can achieve the purpose of tumor suppression. And some studies have found that supplemented thiamine can inhibit the growth and proliferation of tumor cells 72.

​ In disease research, metabolomics can help reveal metabolic abnormalities in the occurrence and development of diseases 73, discover potential biomarkers, and provide important basis for early diagnosis, treatment and prevention of diseases. In this study, mouse serum was analyzed by GC-TOF-MS to analyze L-glutamic acid, glucose-6-phosphate, palmitoleic acid, and linoleic acid involved in energy metabolism, especially glycolysis and fatty acid metabolism. In this study, it was found that guanine, guanine/guanosine and allantoin (metabolites of xanthine) are mainly involved in purine metabolism, and their expression is also up-regulated. Purine metabolism is one of the regulatory pathways involved in lung cancer recurrence models. The results of enrichment analysis also confirmed that purine metabolism is a relevant metabolic pathway. In the comparison of the three dosing groups and the recurrence model group, NVB alone was characterized by metabolites and pathways related to palmitoleic acid and linoleic acid, while the GH group and GH-NVB group were related to purine metabolism pathways.

## Conclusion

In this study, we constructed a mouse lung cancer recurrence model through surgical resection, and used a combination of GH and NVB to treat lung cancer recurrence after surgical resection. Moreover, the combined use of GH and NVB can effectively reduce the side effects of NVB drugs. The results of the study show that the combination of GH and NVB has a good effect in the treatment of lung cancer recurrence after surgical resection. Through the analysis of metabolic pathways, we found that GH and NVB can inhibit postoperative recurrence and improve the quality of life of postoperative patients by improving AP accumulation caused by abnormal purine, leucovorin, and thiamine metabolism. Moreover, we also found in the analysis of metabolites that AP can be used as a biomarker for the detection in the lung cancer treatment. This study can provide a solution for the treatment of lung cancer recurrence, and provide some biomarkers for the treatment of lung cancer recurrence.

## Supplementary Material

Table S1: Dosage schedules of GH and NVB in the administration group, control group and model group. Figure S1: Surgical resection of a mouse lung cancer model. Figure S2: Alanine, aspartate and glutamate metabolism. Figure S3: Valine, leucine and isoleucine biosynthesis. Figure S4: Arginine biosynthesis.

## Figures and Tables

**Table 1:** Differential metabolites between the normal control and recurrence model groups derived from OPLS-DA modeling of GC-TOF/MS data

Class	Compounds	*p*	FC	Model vs. Control	HMDB ID
Amino Acid	Allantoic acid	1.3e-02	1.615	up	HMDB01209
	L-leucine	1.4e-02	0.517	down	HMDB00687
	Ratio of L-valine/alpha-ketoisovaleric acid	1.8e-02	0.405	down	HMDB00883/HMDB00019
	Ratio of oxoglutaric acid/L-glutamic acid	2.7e-02	1.360	up	HMDB00208/HMDB00148
	Ratio of L-glutamine/L-glutamic acid	4.3e-02	0.687	down	HMDB00641/HMDB00148
	L-Proline	4.5e-02	0.604	down	HMDB00162
Carbohydrates	Glucose 6-phosphate	9.4e-03	1.880	up	HMDB01401
Fatty Acids	Palmitic acid	2.9e-02	1.686	up	HMDB00220
	Linoleic acid	3.1e-02	1.658	up	HMDB00673
Nucleotide	Ratio of Guanine/Guanosine	4.3e-02	2.603	up	HMDB00132/HMDB00133
Organic Acids	4-Hydroxybenzoic acid	8.6e-04	2.144	up	HMDB00500
	Malonic acid	7.5e-03	0.315	down	HMDB00691

Note: Variable importance in the projection (VIP) was obtained from the OPLS-DA Model. *P* value and fold-change (FC) values were obtained from Student's t test between the normal and recurrence Model groups. HMDB ID stands for Human Metabolome Database ID.

**Table 2:** Metabolic pathways of distinct compounds in recurrence model group and normal control group.

Pathway	Pathway impact factor	*p*	Up	Down
Neomycin, kanamycin and gentamicin biosynthesis	1.00	1.85e-03	D-Glucose; glucose 6-phosphate	
Valine, leucine and isoleucine biosynthesis	0.25	3.73e-03	Ketoleucine	L-leucine; L-valine
Alanine, aspartate and glutamate metabolism	0.26	5.95e-03	Citric acid; gamma-aminobutyric acid; L-alanine	L-asparagine; L-glutamine
Arginine biosynthesis	0.12	2.01e-02	Citrulline	L-glutamine; urea
Galactose metabolism	0.03	2.67e-02	D-galactose; D-glucose; D-mannose; myoinositol	
Taurine and hypotaurine metabolism	0.33	4.38e-02	Hypotaurine; taurine	Taurine and hypotaurine metabolism
Valine, leucine and isoleucine degradation	0.08	0.091	Ketoleucine; methylmalonic acid	L-leucine; L-valine
Purine metabolism	0.04	0.161	Allantoic acid; guanine	Guanosine; L-glutamine; urea

## References

[1] Minna JD, Roth JA, Gazdar AF (2002). Focus on lung cancer. Cancer cell.

[2] Leiter A, Veluswamy RR, Wisnivesky JP (2023). The global burden of lung cancer: current status and future trends. Nature Reviews Clinical Oncology.

[3] Spyratos D, Zarogoulidis P, Porpodis K, Tsakiridis K, Machairiotis N, Katsikogiannis N (2013). Occupational exposure and lung cancer. Journal of thoracic disease.

[4] Shin SH, Yoon MJ, Kim M, Kim J-I, Lee S-J, Lee Y-S (2007). Enhanced lung cancer cell killing by the combination of selenium and ionizing radiation. Oncology reports.

[5] Ruano-Ravina A, Figueiras A, Barros-Dios J (2000). Diet and lung cancer: a new approach. European journal of cancer prevention.

[6] Rom WN, Hay JG, Lee TC, Jiang Y, Tchou-Wong K-M (2000). Molecular and genetic aspects of lung cancer. American journal of respiratory and critical care medicine.

[7] Naccache J-M, Gibiot Q, Monnet I, Antoine M, Wislez M, Chouaid C (2018). Lung cancer and interstitial lung disease: a literature review. Journal of thoracic disease.

[8] Raghu G, Nyberg F, Morgan G (2004). The epidemiology of interstitial lung disease and its association with lung cancer. British Journal of Cancer.

[9] Rami-Porta R, Wittekind C, Goldstraw P, Committee IAftSoLCS (2005). Complete resection in lung cancer surgery: proposed definition. Lung cancer.

[10] Travis LB, Gospodarowicz M, Curtis RE, Aileen Clarke E, Andersson M, Glimelius B (2002). Lung cancer following chemotherapy and radiotherapy for Hodgkin's disease. Journal of the National Cancer Institute.

[11] Steven A, Fisher SA, Robinson BW (2016). Immunotherapy for lung cancer. Respirology.

[12] Barr Kumarakulasinghe N, Zanwijk Nv, Soo RA (2015). Molecular targeted therapy in the treatment of advanced stage non-small cell lung cancer (NSCLC). Respirology.

[13] Sato T, Watanabe A, Kondo H, Kanzaki M, Okubo K, Yokoi K (2015). Long-term results and predictors of survival after surgical resection of patients with lung cancer and interstitial lung diseases. The Journal of thoracic and cardiovascular surgery.

[14] Suresh R, Ali S, Ahmad A, Philip PA, Sarkar FH (2016). The role of cancer stem cells in recurrent and drug-resistant lung cancer. Lung cancer and personalized medicine: novel therapies and clinical management.

[15] Spiro SG, Gould MK, Colice GL (2007). Initial evaluation of the patient with lung cancer: symptoms, signs, laboratory tests, and paraneoplastic syndromes: ACCP evidenced-based clinical practice guidelines. Chest.

[16] Mokhtari RB, Homayouni TS, Baluch N, Morgatskaya E, Kumar S, Das B (2017). Combination therapy in combating cancer. Oncotarget.

[17] Fisusi FA, Akala EO (2019). Drug combinations in breast cancer therapy. Pharmaceutical nanotechnology.

[18] Wiedmann MW, Mössner J (2013). New and emerging combination therapies for esophageal cancer. Cancer management and research.

[19] Wagner AD, Syn NL, Moehler M, Grothe W, Yong WP, Tai BC (2017). Chemotherapy for advanced gastric cancer. Cochrane database of systematic reviews.

[20] Bunn Jr PA, Kelly K (2000). New combinations in the treatment of lung cancer: a time for optimism. Chest.

[21] Huang X, Huang G, Song H, Chen L (2011). Preconditioning chemotherapy with paclitaxel and cisplatin enhances the antitumor activity of cytokine induced-killer cells in a murine lung carcinoma model. International journal of cancer.

[22] Zhao H-Y, Zhou H-Y, Wang Y-T, Chen W, Qi S-Y, Cao J-L (2016). Assessment on the efficacy and safety of Aidi injection combined with vinorelbine and cisplatin for treatment of advanced nonsmall cell lung cancer. Chinese Medical Journal.

[23] Gong Y, Xu Z, Jin C, Deng H, Wang Z, Zhou W (2018). Treatment of advanced non-small-cell lung cancer with Qi-nourishing essence-replenishing Chinese herbal medicine combined with chemotherapy. Biological Procedures Online.

[24] Chen F, Li X, Wang J, Ma X, Song Z, Sun L (2018). Combination of Ginsenoside H dripping pills and cyclophosphamide improve paraneoplastic syndrome and inhibit postoperative recurrence via the reversion of Th1/Th2 shift. Biomedicine & Pharmacotherapy.

[25] Chen C, Wang Y-S, Zhang E-T, Li G-A, Liu W-Y, Li Y (2021). (20S) Ginsenoside Rh2 exerts its anti-tumor effect by disrupting the HSP90A-Cdc37 system in human liver cancer cells. International Journal of Molecular Sciences.

[26] Ge G, Yan Y, Cai H (2017). Ginsenoside Rh2 inhibited proliferation by inducing ROS mediated ER stress dependent apoptosis in lung cancer cells. Biological and pharmaceutical bulletin.

[27] Guan W, Qi W (2023). Ginsenoside Rh2: a shining and potential natural product in the treatment of human nonmalignant and malignant diseases in the near future. Phytomedicine.

[28] Predina JD, Judy B, Fridlender ZG, Aliperti LA, Madajewski B, Kapoor V (2012). A positive-margin resection model recreates the postsurgical tumor microenvironment and is a reliable model for adjuvant therapy evaluation. Cancer biology & therapy.

[29] Lee JJ, Kong M, Ayers GD, Lotan R (2007). Interaction index and different methods for determining drug interaction in combination therapy. Journal of biopharmaceutical statistics.

[30] Choi WJ, Kim JH, Han GP, Kwon CH, Kil DY (2021). Effects of dietary hatchery by-products on growth performance, relative organ weight, plasma measurements, immune organ index, meat quality, and tibia characteristics of broiler chickens. Animal Bioscience.

[31] Pan L, Qiu Y, Chen T, Lin J, Chi Y, Su M (2010). An optimized procedure for metabonomic analysis of rat liver tissue using gas chromatography/time-of-flight mass spectrometry. Journal of Pharmaceutical and Biomedical Analysis.

[32] Wang J-H, Chen W-L, Li J-M, Wu S-F, Chen T-L, Zhu Y-M (2013). Prognostic significance of 2-hydroxyglutarate levels in acute myeloid leukemia in China. Proceedings of the National Academy of Sciences.

[33] Maćkiewicz A, Ratajczak W (1993). Principal components analysis (PCA). Computers & Geosciences.

[34] Brereton RG, Lloyd GR (2014). Partial least squares discriminant analysis: taking the magic away. Journal of Chemometrics.

[35] Boccard J, Rutledge DN (2013). A consensus orthogonal partial least squares discriminant analysis (OPLS-DA) strategy for multiblock Omics data fusion. Analytica chimica acta.

[36] Ogata H, Goto S, Sato K, Fujibuchi W, Bono H, Kanehisa M (1999). KEGG: Kyoto encyclopedia of genes and genomes. Nucleic acids research.

[37] Wishart DS, Guo A, Oler E, Wang F, Anjum A, Peters H (2022). HMDB 5.0: the human metabolome database for 2022. Nucleic acids research.

[38] Xia J, Sinelnikov IV, Han B, Wishart DS (2015). MetaboAnalyst 3.0—making metabolomics more meaningful. Nucleic acids research.

[39] Namdeo P, Gidwani B, Tiwari S, Jain V, Joshi V, Shukla SS (2023). Therapeutic potential and novel formulations of ursolic acid and its derivatives: An updated review. Journal of the Science of Food and Agriculture.

[40] Ren Y, Qian Y, Zhang Q, Li X, Li M, Li W (2024). High LGALS3 expression induced by HCP5/hsa-miR-27b-3p correlates with poor prognosis and tumor immune infiltration in hepatocellular carcinoma. Cancer Cell International.

[41] Hoy H, Lynch T, Beck M (2019). Surgical Treatment of Lung Cancer. Critical care nursing clinics of North America.

[42] Kelsey CR, Marks LB, Hollis D, Hubbs JL, Ready NE, D'Amico TA (2009). Local recurrence after surgery for early stage lung cancer: an 11-year experience with 975 patients. Cancer.

[43] Tang X-P, Tang G-D, Fang C-Y, Liang Z-H, Zhang L-Y (2013). Effects of ginsenoside Rh2 on growth and migration of pancreatic cancer cells. World Journal of Gastroenterology: WJG.

[44] Kim JH, Yi Y-S, Kim M-Y, Cho JY (2017). Role of ginsenosides, the main active components of Panax ginseng, in inflammatory responses and diseases. Journal of ginseng research.

[45] Ratan ZA, Youn SH, Kwak Y-S, Han C-K, Haidere MF, Kim JK (2021). Adaptogenic effects of Panax ginseng on modulation of immune functions. Journal of ginseng research.

[46] Gao X-Y, Liu G-C, Zhang J-X, Wang L-H, Xu C, Yan Z-A (2022). Pharmacological properties of ginsenoside Re. Frontiers in pharmacology.

[47] Sun M, Ye Y, Xiao L, Duan X, Zhang Y, Zhang H (2017). Anticancer effects of ginsenoside Rg3. International journal of molecular medicine.

[48] Peng Z, Wu WW, Yi P (2021). The efficacy of ginsenoside Rg3 combined with first-line chemotherapy in the treatment of advanced non-small cell lung cancer in China: a systematic review and meta-analysis of randomized clinical trials. Frontiers in Pharmacology.

[49] Keller SM, Adak S, Wagner H, Herskovic A, Komaki R, Brooks BJ (2000). A randomized trial of postoperative adjuvant therapy in patients with completely resected stage II or IIIA non-small-cell lung cancer. New England Journal of Medicine.

[50] Buffoni L, Dongiovanni D, Barone C, Fissore C, Ottaviani D, Dongiovanni V (2006). Fractionated dose of cisplatin (CDDP) and vinorelbine (VNB) chemotherapy for elderly patients with advanced non-small cell lung cancer: Phase II trial. Lung Cancer.

[51] Toschi L, Finocchiaro G, Bartolini S, Gioia V, Cappuzzo F (2005). Role of gemcitabine in cancer therapy. Future Oncol.

[52] Abu Samaan TM, Samec M, Liskova A, Kubatka P, Büsselberg D (2019). Paclitaxel's mechanistic and clinical effects on breast cancer. Biomolecules.

[53] Yardley DA (2013). nab-Paclitaxel mechanisms of action and delivery. Journal of Controlled Release.

[54] Adjei AA (2004). Pemetrexed (ALIMTA), a novel multitargeted antineoplastic agent. Clinical Cancer Research.

[55] Baldwin E, Osheroff N (2005). Etoposide, topoisomerase II and cancer. Current Medicinal Chemistry-Anti-Cancer Agents.

[56] Gasparini G, Caffo O, Barni S, Frontini L, Testolin A, Guglielmi RB (1994). Vinorelbine is an active antiproliferative agent in pretreated advanced breast cancer patients: a phase II study. Journal of clinical oncology.

[57] Qin R-S, Zhang Z-H, Zhu N-P, Chen F, Guo Q, Hu H-W (2018). Enhanced antitumor and anti-angiogenic effects of metronomic Vinorelbine combined with Endostar on Lewis lung carcinoma. BMC cancer.

[58] Liu Y, Li G, Ning J, Zhao Y (2024). Unveiling the experimental proof of the anticancer potential of ginsenoside Rg3. Oncology Letters.

[59] Zhang B, Pei W, Cai P, Wang Z, Qi F (2022). Recent advances in Chinese patent medicines entering the international market. Drug Discoveries & Therapeutics.

[60] Ma J, Waxman DJ (2008). Combination of antiangiogenesis with chemotherapy for more effective cancer treatment. Molecular cancer therapeutics.

[61] Gilad Y, Gellerman G, Lonard DM, O'malley BW (2021). Drug combination in cancer treatment—From cocktails to conjugated combinations. Cancers.

[62] Mignani S, Bryszewska M, Klajnert-Maculewicz B, Zablocka M, Majoral J-P (2015). Advances in combination therapies based on nanoparticles for efficacious cancer treatment: an analytical report. Biomacromolecules.

[63] Dasari S, Njiki S, Mbemi A, Yedjou CG, Tchounwou PB (2022). Pharmacological effects of cisplatin combination with natural products in cancer chemotherapy. International Journal of Molecular Sciences.

[64] Prakash O, Kumar A, Kumar P (2013). Anticancer potential of plants and natural products. Am J Pharmacol Sci.

[65] Oh M, Choi Y, Choi S, Chung H, Kim K, Kim SI (1999). Anti-proliferating effects of ginsenoside Rh2 on MCF-7 human breast cancer cells. International journal of oncology.

[66] Nakata H, Kikuchi Y, Tode T, Hirata J, Kita T, Ishii K (1998). Inhibitory effects of ginsenoside Rh2 on tumor growth in nude mice bearing human ovarian cancer cells. Japanese Journal of Cancer Research.

[67] Wishart DS, Mandal R, Stanislaus A, Ramirez-Gaona M (2016). Cancer metabolomics and the human metabolome database. Metabolites.

[68] Aboud OA, Weiss RH (2013). New opportunities from the cancer metabolome. Clinical chemistry.

[69] Kaushik AK, DeBerardinis RJ (2018). Applications of metabolomics to study cancer metabolism. Biochimica et Biophysica Acta (BBA)-Reviews on Cancer.

[70] Meng H, Cao Y, Qin J, Song X, Zhang Q, Shi Y (2015). DNA methylation, its mediators and genome integrity. International journal of biological sciences.

[71] Rapala-Kozik M (2011). Vitamin B1 (thiamine): a cofactor for enzymes involved in the main metabolic pathways and an environmental stress protectant. Advances in botanical research: Elsevier.

[72] Liu X, Montissol S, Uber A, Ganley S, Grossestreuer AV, Berg K (2018). The effects of thiamine on breast cancer cells. Molecules.

[73] Zhang A, Sun H, Wang X (2012). Serum metabolomics as a novel diagnostic approach for disease: a systematic review. Analytical and bioanalytical chemistry.

