# Supplementary material for: Metabolomics-based combination of GH and NVB in the treatment of NSCLC lung cancer recurrence
Source: J Cancer. 2025 Jan 1;16(1):265–78. doi: 10.7150/jca.102722 (PMC11660129; doi:10.7150/jca.102722)
Supplement: Supplementary file 1 — Table S1: Dosage schedules of GH and NVB in the administration group, control group and model group. Figure S1: Surgical resection of a mouse lung cancer model. Figure S2: Alanine, aspartate and glutamate metabolism. Figure S3: Valine, leucine and isoleucine biosynthesis. Figure S4: Arginine biosynthesis. [file jcav16p0265s1.pdf]

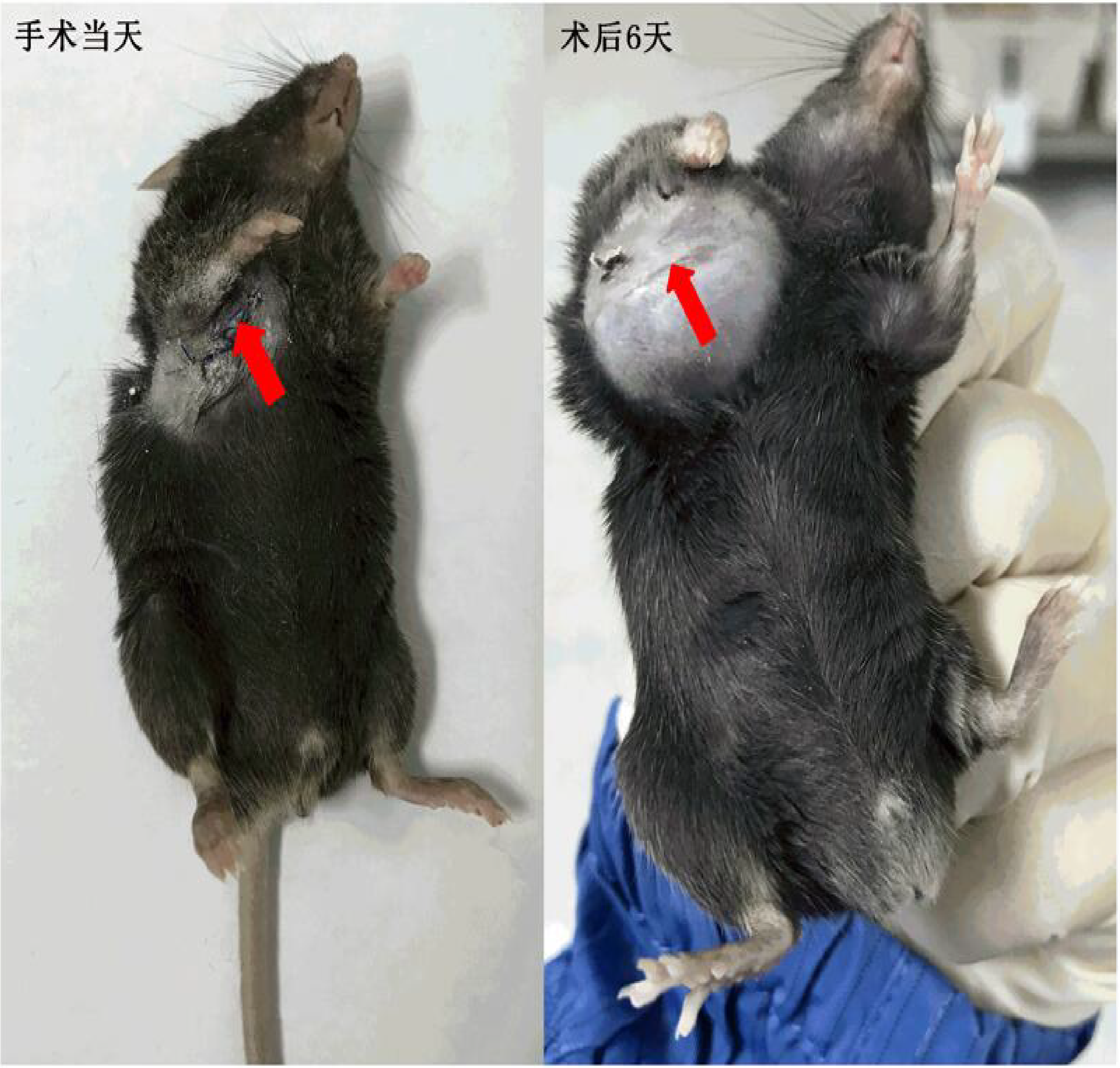
手术当天
术后6天

Neomycin, kanamycin and gentamicin biosynthesis

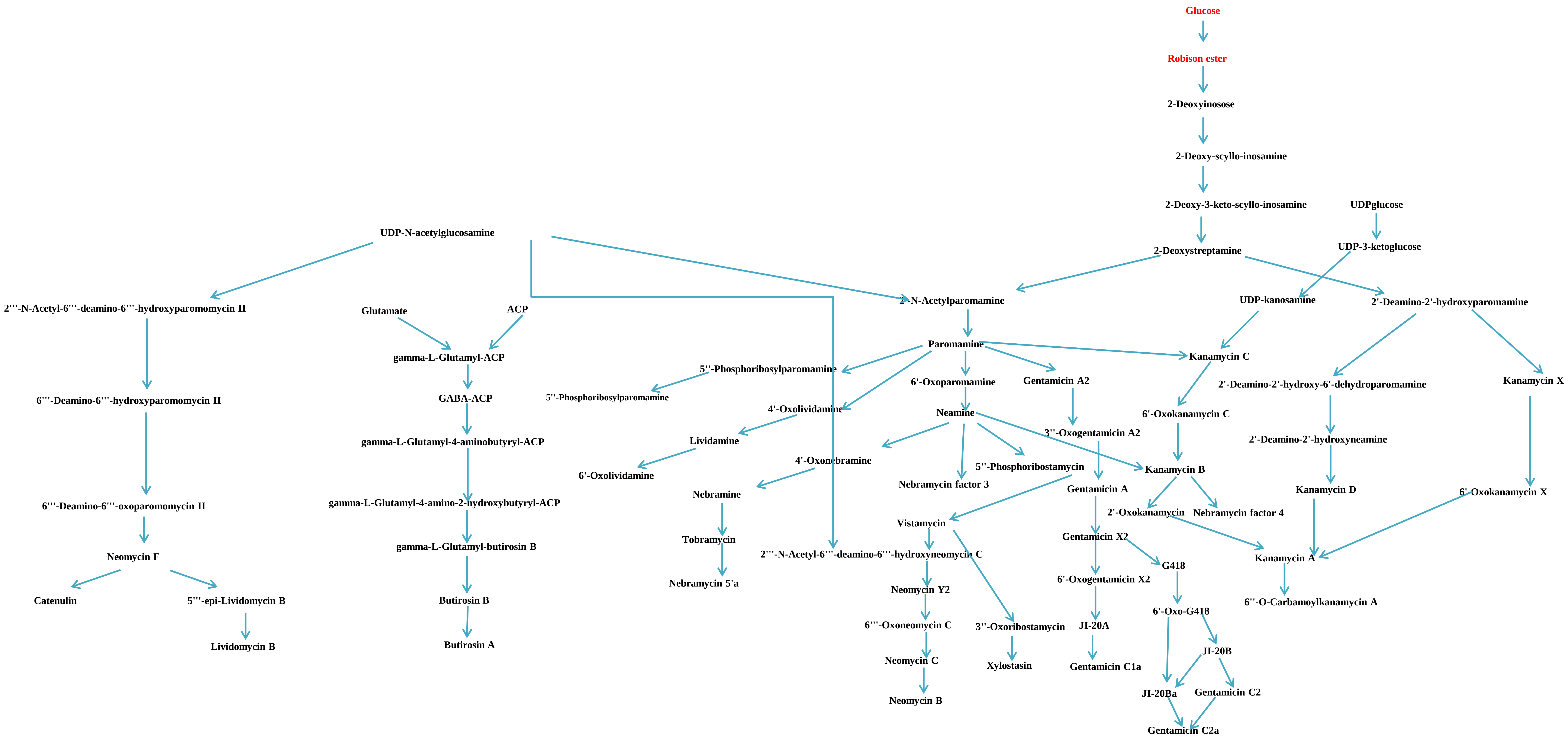

Valine, leucine and isoleucine biosynthesis

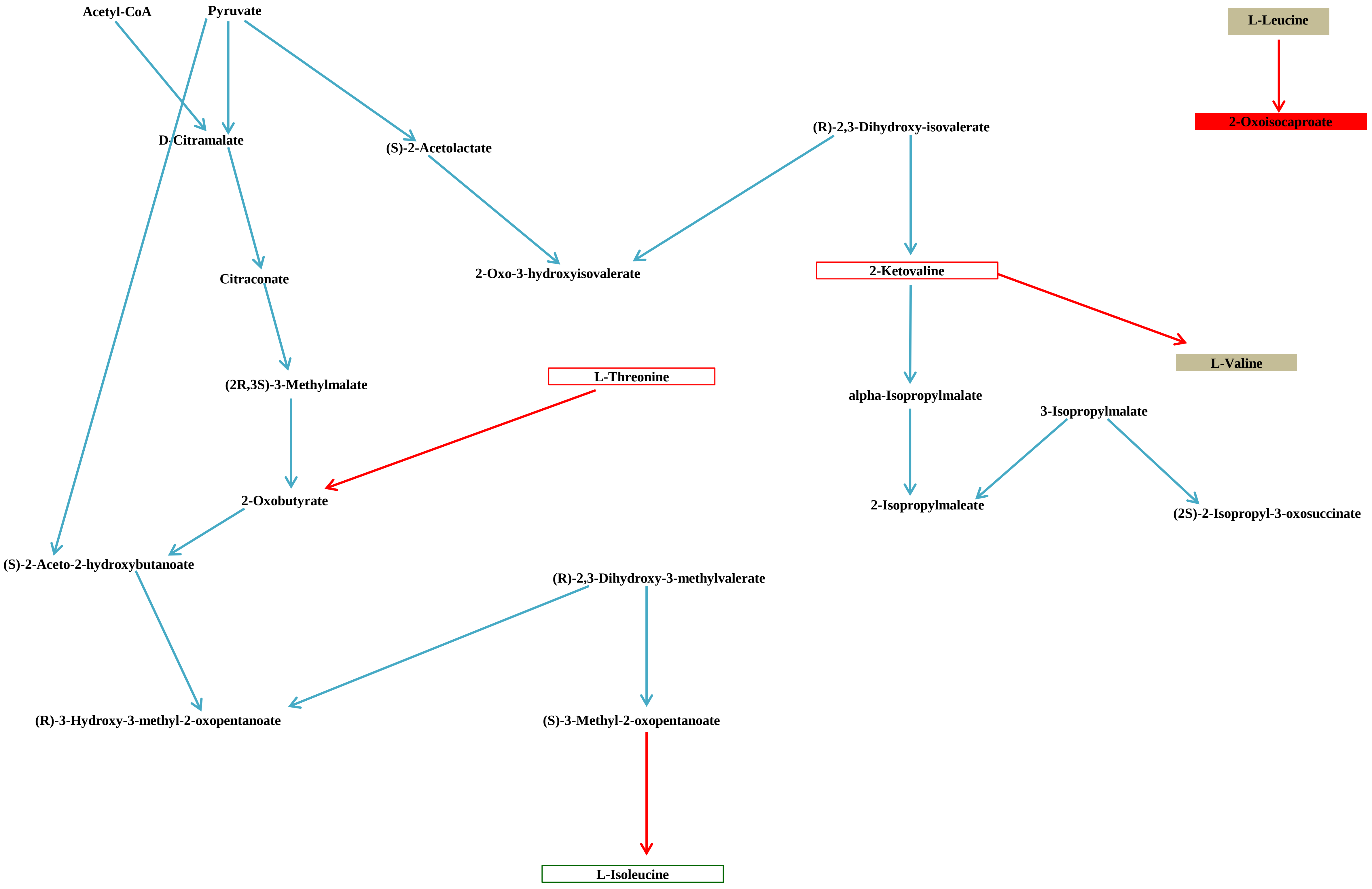

Alanine, aspartate and glutamate metabolism

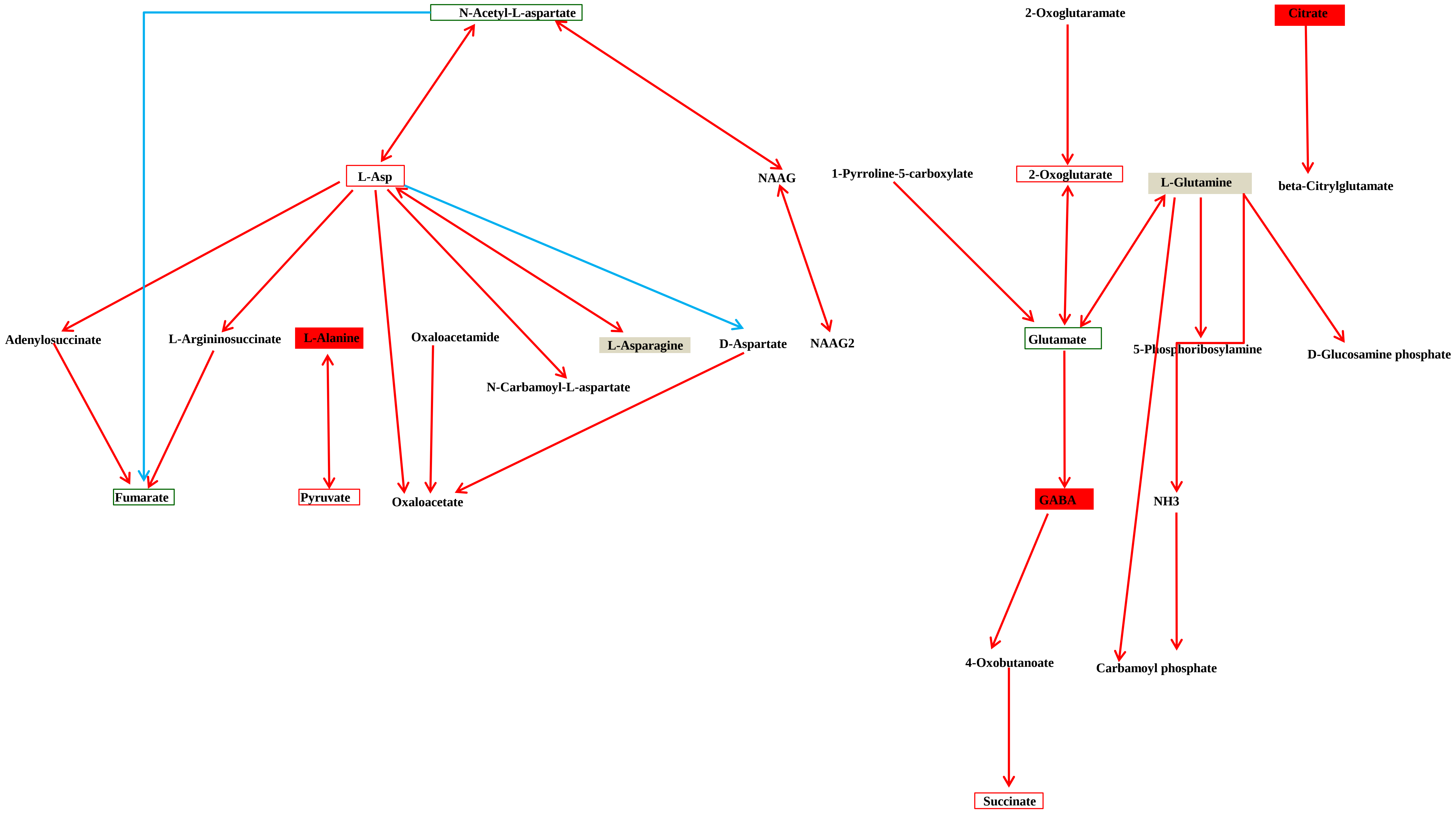

Arginine biosynthesis

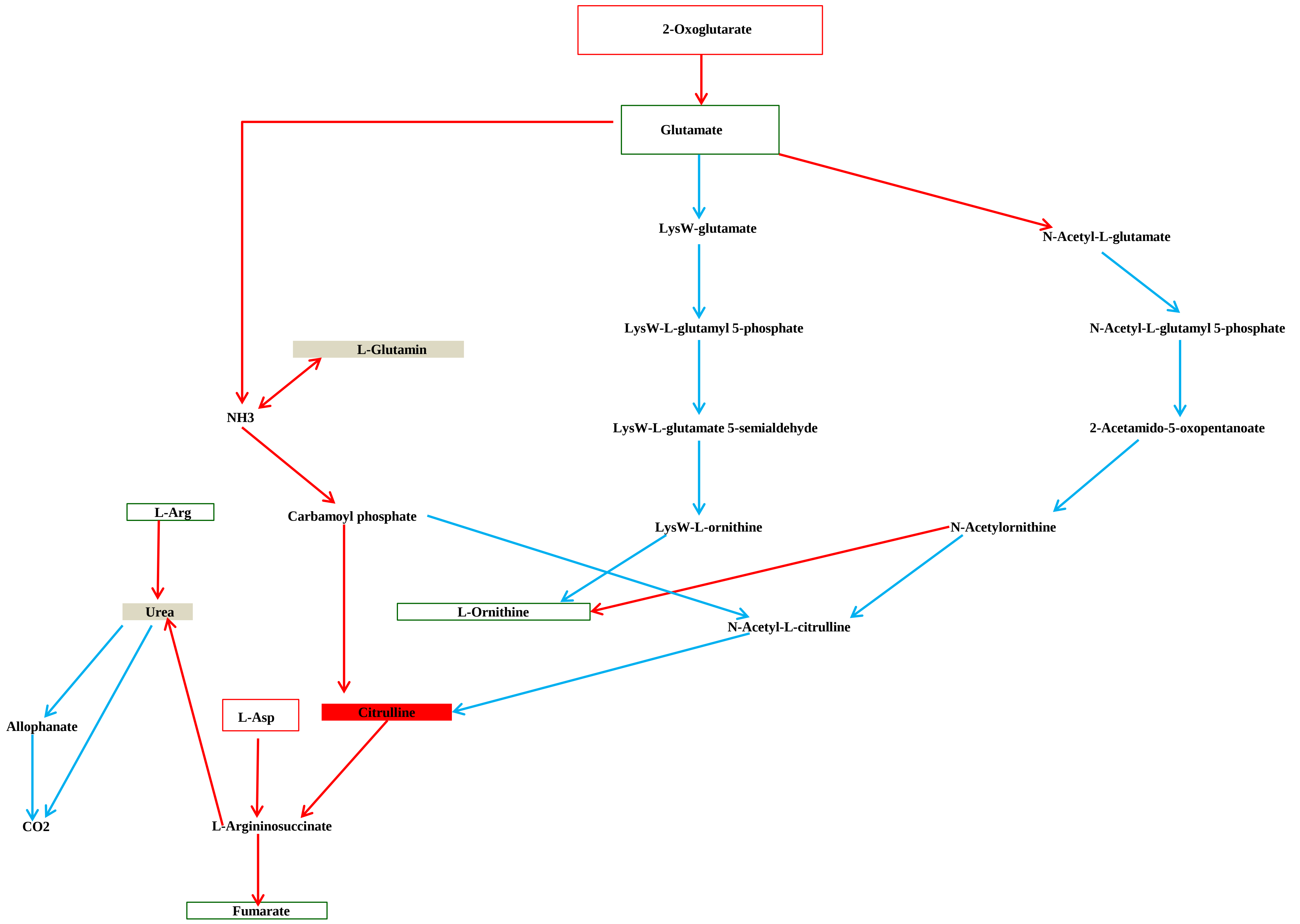

Table S1 Groups and therapeutic regimens of GH and NVB in the recurrence Model

Group	Number	Dosage
Control	8	0.5% CMC-Na (0.1 ml/10 g, per day), saline(0.1 ml/10 g, per week)
Model	8	0.5% CMC-Na (0.1 ml/10 g, per day), saline(0.1 ml/10 g, per week)
GH	8	36 mg/kg Gh in 0.5% CMC-Na (0.1 ml/10 g, per day), saline(0.1 ml/10 g, per week)
NVB	8	0.5% CMC-Na (0.1 ml/10 g, per day), 4.15 mg/kg NVB in saline(0.1 ml/10 g, per week)
GH-NVB	8	36 mg/kg Gh in 0.5% CMC-Na (0.1 ml/10 g, per day), 4.15 mg/kg NVB in saline(0.1 ml/10 g, per week)
